# Assessment of short-term changes in street dust pollution with heavy metals in Lublin (E Poland)—levels, sources and risks

**DOI:** 10.1007/s11356-019-06496-x

**Published:** 2019-10-30

**Authors:** Wojciech Zgłobicki, Małgorzata Telecka, Sebastian Skupiński

**Affiliations:** Faculty of Earth Sciences and Spatial Management, Maria-Curie Sklodowska University, Krasnicka 2D, 20-718 Lublin, Poland

**Keywords:** Air quality, Trace metals, Geochemical indices, Pollution changes, Road dust, Urban environment

## Abstract

Street dust forms as a result of the interaction of the atmosphere, lithosphere (pedosphere) and anthroposphere and can be regarded as an index of the condition of the environment in urban areas. At the end of the twentieth century, there was a significant decrease in heavy metal emissions in Europe, but not so intensive in Poland. The question arises: Is the intensity of pollution still decreasing? The study objective was to assess changes in street dust pollution with heavy metals in Lublin (E Poland) in the years 2013 and 2018. The sample collection sites (68) were located within streets with a varying intensity of motor traffic. Cd, Cr, Cu, Ni, Pb and Zn concentrations were determined in two dust fractions, 63–200 μm and < 63 μm, by means of an X-ray fluorescence spectrometer. The levels of street dust pollution with heavy metals, expressed both in absolute concentrations and geochemical indices, were lower in 2018 than those in 2013. The clearest decrease of concentration levels occurred within the main roads, in the 63–200 μm fraction for Cu and Cd, and in both fractions for Pb. The mean concentrations of the investigated metals, normalised to the background values, are in the following order for both fractions in 2013 and 2018: Zn > Cd > Cu > Cr > Pb > Ni. Metals form the following order for *I*_geo_ and EF: Zn > Cd > Cu > Pb > Cr > Ni. This order is slightly different for the ecological risk factor: Cd > Cu > Pb > Zn > Cr > Ni. In general, street dust in Lublin does not show contamination with Cr, Ni and Pb. *I*_geo_ and EF indices show moderate levels of pollution with Cu, Cd and Zn.

## Introduction

Re-suspended street dust is a major source of air and soil pollution in cities (Wong et al. 2006; Thorpe and Harrison 2008; Charlesworth et al. 2011; Karimian Torghabeh et al. 2019). It consists of mineral and organic particles originating from natural sources, industrial emitters and motor vehicle traffic. Quartz and feldspars are the main mineral components of street dust. It also contains various kinds of toxic compounds, including heavy metals such as Cd, Cu, Pb and Zn. Their main sources are car exhaust emission and the wear and tear of various vehicle parts (tyres, brake discs), road pavements or the overhead lines (used by trams, trolleybuses and trains). Heavy metals can also originate from fluids released from vehicles onto the surface of the streets. Street dust also contains mineral particles from soils adjoining roads (Bilos et al. 2001; Manno et al. 2006; Amato et al. 2011).

Street dust forms as a result of the interaction of the atmosphere, lithosphere (pedosphere) and anthroposphere; it can impact the biosphere and human beings. Therefore, it can be regarded as an index of the condition of the environment in urban areas (Shi et al. 2010; Pan et al. 2017). Since street dust is removed from streets, the results of repeated investigations allow assessing the variation of the degree of environmental pollution and indicating its general trends (Han et al. 2007; Lu et al. 2017). Pollutants accumulating in street dust are exceptionally hazardous due to the possibility of its re-suspension and occurrence at low height, in the living zone of humans, plants and animals. This entails the risk of heavy metal penetration into the human body, which is a great threat to human health (Aelion et al. 2008; Zheng et al. 2010; Charlesworth et al. 2011; Gope et al. 2017). Since heavy metals have a bioaccumulation potential, with time, their levels in the human body can be higher than in the environment (Padoan et al. 2017). Most of the contaminants in dust emitted as a result of motor vehicle transport are deposited within 50 m from roads (Ordóñez et al. 2003; Plak et al. 2010).

Studies conducted in cities around the world indicate increased concentrations of trace metals in street dust (e.g. Sezgin et al. 2003; Ahmed and Ishiga 2006; Han et al. 2006; Al-Khashman 2004, 2007; Shi and Wang 2013; Chen et al. 2019). Such increased levels of concentration are observed in particular for lead, cadmium, copper, chromium and nickel. Investigations focus on big cities characterised by a high intensity of car traffic and presence of industrial emitters (Du et al. 2013; Li and Liu 2001; Sezgin et al. 2003; Zhao et al. 2015). These studies usually present the results of research determining the pollution level based on a single sampling. Seasonal collection of street dust is also sometimes applied (Zhang et al. 2013). Attention is also drawn to the fact that finer street dust fractions pose an even greater threat because of how easily they can penetrate the respiratory tract, and due to higher concentrations of toxic metals (Han et al. 2008; Shi and Lu 2018).

Recent research conducted to date points to a diverse health risk related to the content of heavy metals in road dust. In the review of studies regarding the area of China, Hou et al. (2019) state that none of the hazard index values exceeded the safe level (except one city). Cr was reported as a potential health risk for children in Dhaka (Safiur Rahman et al. 2019). On the other hand, heavy metals occurring in road dust in industrial and mining areas are a serious threat to human health (Tian et al. 2019). The impact of particle size—with fine particles posing a more intense threat—is underlined in all the papers mentioned above.

During the past three decades, much efforts have been done to reduce emissions of heavy metals in Europe (Harmens et al. 2013). Especially in the period from 1990 to 2009 and in a case of Pb and Cd (European Environment Agency, Heavy metals emissions, 2018). Additionally, according to European Environment Agency, road transport is one of Europe’s main sources of air pollution. However, in Poland, decrease of emissions was the smallest in Europe. This is due to the widespread use of coal as fuel in power plants and households, as well as the growing number of cars. The following question therefore arises: Does the decrease in the content of heavy metals in the air and what is consequently in street dust still occur in Poland?

The study objective was to assess the degree of street dust enrichment in heavy metals (Cd, Cu, Cr, Ni, Pb and Zn) in the area of city of Lublin (E Poland) for the period 2013–2018. The metals chosen for analysis are commonly regarded as indices of environmental pollution linked to motor vehicle traffic and industrial operations (De Miguel et al. 1997; Kabata-Pendias and Pendias 1999; Charlesworth et al. 2011). Investigations of street dust pollution in Lublin were based on the analysis of material sampled from the same location at an interval of 5 years. The measurements were carried out for two fractions, 63–200 μm and < 63 μm. It allows to determine trends in the condition of the environment in the city and to assess the enrichment of the finest fraction in toxic metals. Our study will make it also to estimate short-lasting changes in heavy metal pollution threatening residents’ health in medium-sized cities as well as the causes of these changes. It is one of the first studies of this kind in Poland. It should also be stressed that studies on street dust pollution are usually conducted in large and very large cities. On the other hand, Poland is considered a country with a high degree of air pollution and, therefore, the investigation of heavy metal content in street dust is particularly important here.

## Study area and method

Lublin is located in the north-western part of the Lublin Upland, eastern Poland (51°08′23.31″–51°17′47.61″N, 22° 27′15.41″–22° 40′24.75″E). The city covers an area of nearly 150 km^2^, inhabited by almost 350 thousand residents. Lublin is now an academic, administrative and service centre, and no longer has an industrial character. Some small industrial areas are located in the eastern part of the city, but they are not a source of significant industrial pollution because of the predominance of westerly winds. First and foremost, not very large food, pharmaceutical, chemical and construction industry enterprises operate here. Studies carried out thus far indicate a relatively low level of the pollution of soils in Lublin with heavy metals (Pasieczna 2003; Plak et al. 2010).

**Fig. 1:**
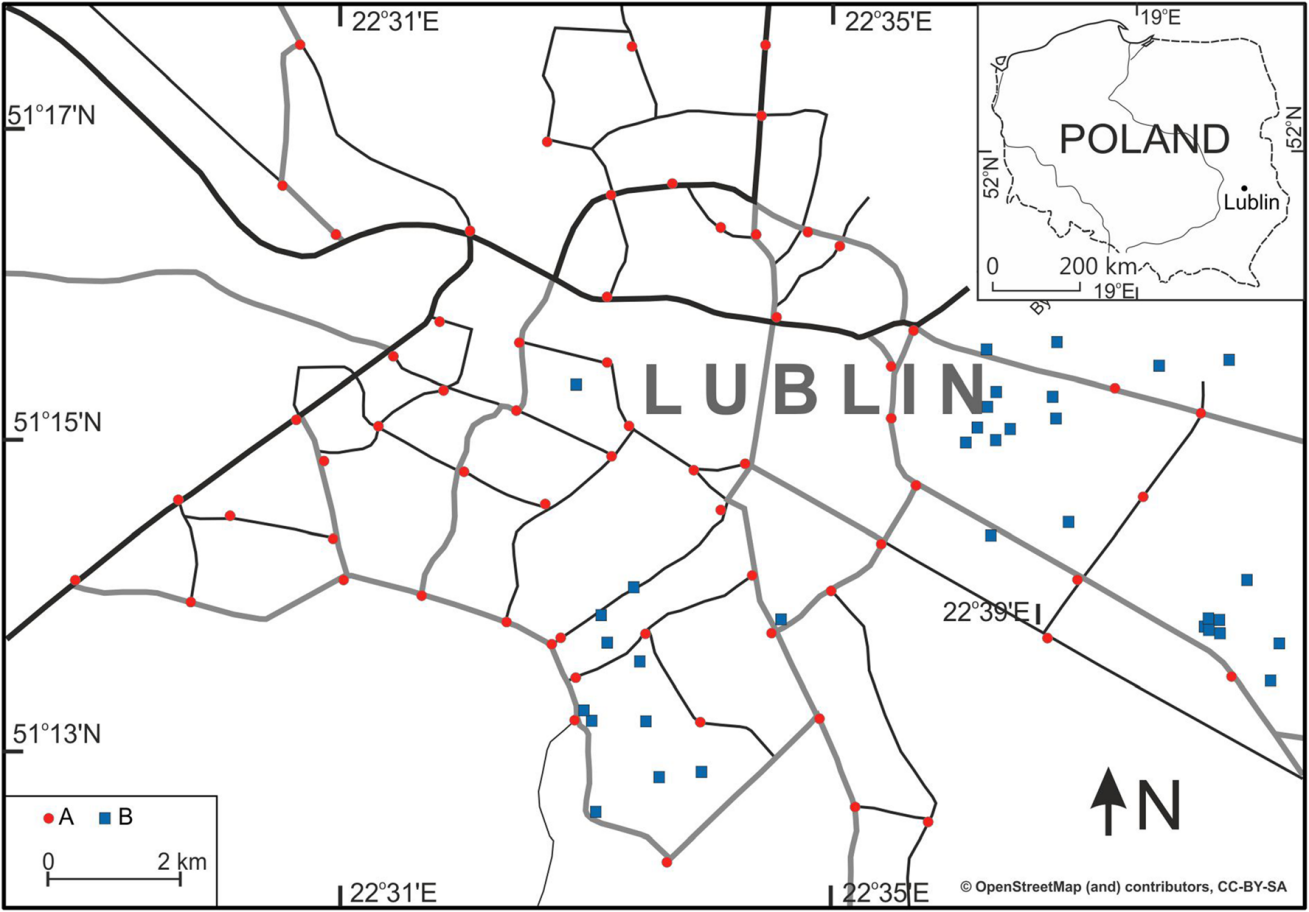
Location of sample collection points in relation to Lublin’s road network. (A) Sample collection points. (B) Small industrial plants

The sample collection points were located within transport routes with a varying intensity of motor vehicle traffic. The analysed samples were collected from a total of 68 sites (Fig. 1) located in residential districts, retail and service centres and industrial areas. The study material originated from the street pavements and was sampled in places of dust accumulation along the edges of the roadway, close to street crossings. The material was sampled twice: first in April 2013 and then in April 2018. The sample collection method used is common in investigations of this kind (Han et al. 2006; Lanzerstorfer 2018). The dust was sampled after a period of a few consecutive days without precipitation. The material was swept up with a polyethylene brush into plastic bags, then dried at room temperature and subjected to preliminary sifting through a 1-mm sieve in order to remove macroremains. Then, the samples were sieved through a nylon sieve with a diameter of 200 μm and subsequently 63 μm. Two dust fractions, (i) 63–200 μm and (ii) < 63 μm, were obtained in this way.

The material for analysis was ground in a zirconia ball mill (Micro Mill) and then prepared in the form of pressed tablets (10 g sample and 1 g wax). Analyses of Cd, Cr, Cu, Ni, Pb and Zn concentrations were carried out with an energy dispersive X-ray fluorescence spectrometer (Epsilon5 Panalytical) (Safiur Rahman et al. 2019). Metal content was measured in each sample three times, and the average of the measured values was recorded as the final result. The accuracy of the method was verified using reference material NCS DC 73385 with certified metal content: Cd = 2 mg kg^−1^, Cr = 370 mg kg^−1^, Cu = 40 mg kg^−1^, Ni = 64 mg kg^−1^, Pb = 58 mg kg^−1^, Zn = 210 mg kg^−1^. The measurement error varied from 3 to 5% and was similar to values presented for the method by other authors (Safiur Rahman et al. 2019). The measurement results for 10 samples of uncontaminated soils collected in the immediate vicinity of the city were used to determine the natural metal content levels in the environment. The geochemical background values were determined separately for both fractions: 63–200 μm and < 63 μm.

The variation of heavy metal concentration in street dust was analysed with descriptive statistics (average, minimum, maximum, coefficient of variation). In order to determine the degree of enrichment and threat to the environment, the following geochemical indices were used: (i) geoaccumulation index (*I*_geo_), (ii) enrichment factor (EF), (iii) index of ecological risk (*Er*_*i*_) and (iv) potential ecological risk index (RI) (Müller 1979; Håkanson 1980; Ergin et al. 1991; Zhao et al. 2015). The indices above allow distinguishing metals from anthropogenic sources and assessing the influence of human activity on the environment. Principal component analysis (PCA) and cluster analysis (CA) were used to determine the correlations between metal concentrations, which enabled indicating their source of origin (Han et al. 2006; Tokalıoğlu and Kartal 2006).

The geoaccumulation index is defined by the following formula:

$$ {I}_{\mathrm{geo}}={\log}_2\left(\raisebox{1ex}{${C}_n$}\!\left/ \!\raisebox{-1ex}{$1.5\ {C}_{\mathrm{ref}}$}\right.\right) $$

*C*_*n*_ denotes the concentration of the metal, and *C*_ref_ value of the background. Six categories of pollution are distinguished: unpolluted (*I*_geo_ < 0), unpolluted to moderately polluted (0–1), moderately polluted (1–2), moderately to strongly polluted (2–3), highly polluted (3–4), highly to extremely polluted (4–5) and extremely polluted (> 5) (Müller 1979):

Enrichment factor is calculated with the following formula:

$$ \mathrm{EF}=\frac{C_n/{C}_{\mathrm{ref}}}{B_n/{B}_{\mathrm{ref}}} $$

*C*_*n*_ is the metal concentration in a sample, *C*_ref_ background value for the metal, *B*_*n*_ reference metal concentration in sample (Mn was used as a reference element) and *B*_ref_ background value for reference metal. Five categories of pollution were distinguished: minimal (EF < 2), moderate (2–5), significant (5–20), very high (20–40) and extreme (> 40) (Ergin et al. 1991).

Index of ecological risk factor is defined as follows:

$$ {Er}_i={T}_i\times \frac{C_n}{C_{\mathrm{ref}}} $$

*T*_*i*_ is the toxic-response factor for the metal. Håkanson (1980) provides the following values for this index: Cd, 30; Cr, 2; Cu, 5; Ni, 5; Pb, 5; Zn 1. Five categories of pollution are distinguished: low (< 40), moderate (40–80), considerable (80–160), high (160–320) and very high (> 320).

Potential ecological risk (RI) is defined as the sum of the index of ecological risk factors (*Er*_*i*_) for specific metals in a sample. Four categories of the index are distinguished (Håkanson 1980): low (< 150), moderate (150–300), considerable (300–600) and high (> 600).

## Results

### Concentrations

Zn was the element with the greatest enrichment in relation to the geochemical background for both fractions and on both measurement dates. On average, its concentration in street dust was 4.5–8.8 times higher than in natural conditions (in soil). In the case of Cd, this index ranged from 3.8 to 7.8. It was similar for Cu: 3.5 to 6.7. For Pb, the index was 1.3 to 2.9. The smallest enrichment was found for Cr (1.4–1.8) and Ni (1.2–1.7). For all elements except Cr, the enrichment factors were higher in 2018 and for the finer fraction. The maximum enrichment levels found for individual samples (< 63 μm fraction, year 2013) were as follows: Cd, 13; Cr, 6.5 (2018, 63–200 μm fraction); Cu, 30; Ni, 2.5; Pb, 8; Zn, 16 (Tables 1 and 2)

**Table 1:** Content of heavy metals in street dust (63–200 μm) in Lublin (mg kg^−1^)

	Cd	Cr	Cu	Ni	Pb	Zn
	2013/2018					
Mean value	5.1/3.8	84.4/120	80.1/57.7	16.2/11.9	43.5/25.4	238.8/177.8
Min. value	2.9/1.6	33.9/62	20.8/16.6	5.1/6.1	21.1/13.1	71.2/71.1
Max. value	13.8/5.7	174.2/324	337.7/178.9	22.6/20.5	129.7/50.9	578.6/625.3
Standard deviation	1.7/0.6	23.3/32.1	69.2/37.0	3.9/3.2	16.4/8.0	94.6/82.5
Variation coefficient (VC) (%)	33/15	26/27	78/64	22/27	36/31	34/46
Geochemical background	1.0	50.0	16.5	9.5	19.6	39.4

**Table 2:** Content of heavy metals in street dust (< 63 μm) in Lublin (mg kg^−1^)

	Cd	Cr	Cu	Ni	Pb	Zn
	2013/2018					
Mean value	6.3/5.5	108.5/112	114.9/120.6	21.4/17.1	62.0/46.6	364.4/296.2
Min. value	4.1/4.0	69.8/53	43.2/26.3	10.9/9.1	32.3/29.5	127.6/99.2
Max. value	10.4/7.9	153.8/274	485.3/353	31.9/26.9	173.6/94.8	618.0/587.3
Standard deviation	1.5/0.9	18.6/43.8	60.5/82.3	4.2/3.6	21.8/14.1	117.6/103.7
Variation coefficient (VC) (%)	23/16	17/39	52/68	19/21	35/30	32/35
Geochemical background	0.8	60.0	17.8	12.4	21.4	41.3

**Fig. 2:**
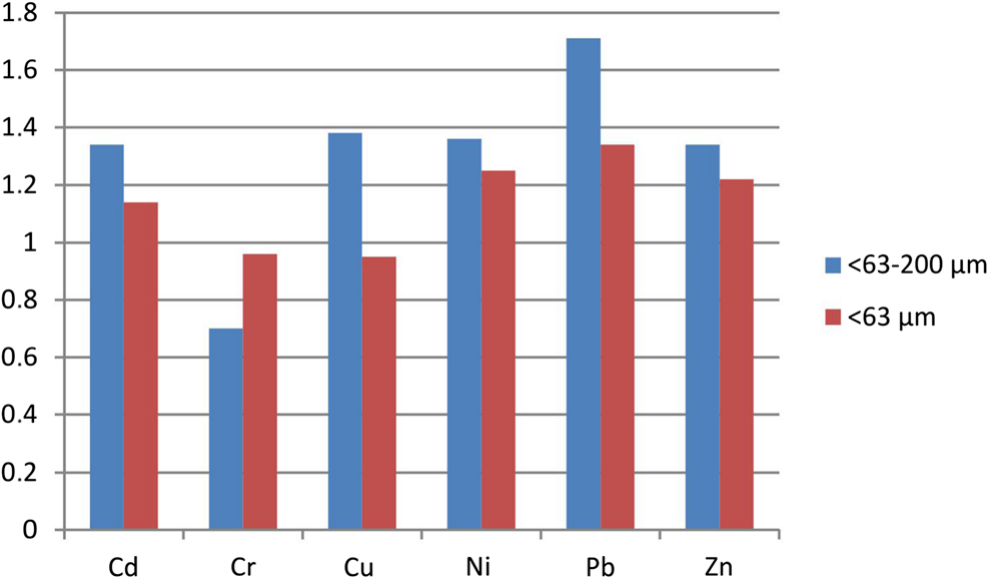
Ratio between the content of the metals under study in 2013 and 2018 in the 63–200 μm and < 63 μm fractions

A general decrease in concentrations of all metals except Cr was found in the street dust samples in the 2013–2018 period (Fig. 2); the decrease was distinctly stronger for the coarser fraction. It was the most visible for Pb: the mean decrease observed in the 63–200 μm fraction reached 41% (20–60% for the individual samples). For Cd, Cu, Ni and Zn, the mean decrease was 25–28%. The mean Cr content in 2018 was 40% higher than in 2013. The biggest decrease, by 25%, was observed for Pb also in the finer fraction (< 63 μm). For Ni and Zn, the decrease was 20% and 18%, respectively, while no essential changes were recorded for Cr and Cu.

**Fig. 3:**
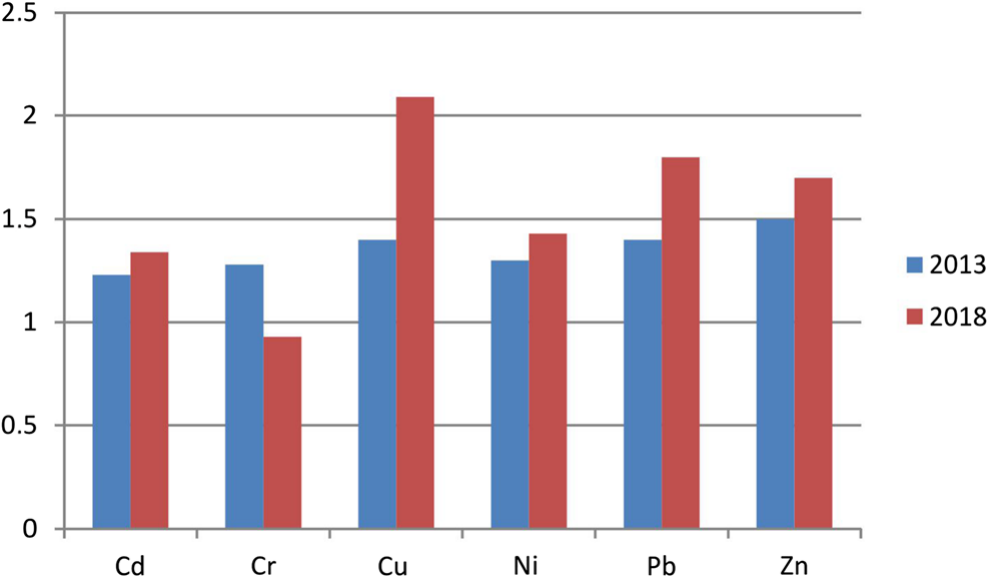
Ratio between the content of the metals under study in the 63–200 μm and < 63 μm fractions in 2013 and 2018

The mean concentrations of metals in the < 63 μm fraction were distinctly higher for all elements except for Cr (Fig. 3). The biggest difference occurred in the case of Cu whose mean content in the finer fraction in 2018 was twice as high as in the coarser fraction. In the same year, this ratio was 1.8 for Pb and 1.7 for Zn, while in 2013, it was lower: 1.4 and 1.5, respectively. Even smaller differences were found in the case of Ni (1.3–1.4) and Cd (1.2–1.4).

In comparison to 2013, a distinct decrease of minimal concentrations occurred in 2018 in the < 63 μm fraction in the case of Cu (39%), Cr (24%) and Zn (20%). No changes were observed for the other elements. In the 63–200 μm fraction, the situation was more varied: for Cd, Pb and Cu, minimal concentration levels fell by 44, 38 and 22%, respectively, while they increased by 82 and 19% for Cr and Ni, respectively.

The maximum concentration levels were also lower in 2018. The largest differences were recorded in the 63–200 μm fraction: a decrease in the case of Cu (63%), Pb (60%) and Cd (58%), and an 86% increase for Cr, while basically no changes occurred for Ni and Zn. In the finer fraction (< 63 μm), the biggest decrease was observed for Pb (45%) and Cd (43%). The maximum Cr content rose by 79%. In the case of the other elements, the changes were small, by a maximum of 15% (Ni).

Variation coefficients were usually slightly higher for the 63–200 μm fraction (Table 1). The highest coefficient values were found for Cu (high variation); they were distinctly lower for Pb, Zn and Cd (medium variation), and the lowest for Cr and Ni (low variation). Different trends can be observed in the changes. The index fell considerably in the case of Cd (in both fractions) while it increased for Cr (fraction < 63 μm); in the case of Cu, it decreased in the 63–200 μm fraction and increased in the finer fraction; a slight decrease occurred for Pb; there were no changes for Ni and Zn.

### Geochemical indices

#### Geoaccumulation index

The 63–200 μm fraction showed no contamination with Ni, a low level of pollution with Cr and Pb, moderate with Cd and a moderate to high level of pollution with Cu and Zn. For all elements except Cr, the pollution level was higher in 2013. The most distinct decrease in 2018 was found for Zn. In the case of the finer fraction (< 63 μm), a low level of pollution was found for Cr and Ni, low to moderate for Pb and moderate to high for Cd, Cu and Zn. Some samples (up to 25%) had a high level of pollution with Cu and Zn. Also in this case, the pollution level was higher in 2013. Only in the case of Cu, a greater number of samples with a high level of pollution was found in 2018 (11, compared with 4 in 2013). A considerable decrease of pollution with Cd and Pb occurred (Tables 3, 4 and 5).

**Table 3:** Mean values and pollution classes according to geoaccumulation index (*I*_geo_), enrichment factor (EF) and ecological risk (ER) index

	Cd	Cu	Cr	Ni	Pb	Zn
	2013/2018					
63–200 μm						
*I*_geo_	1.8/1.4 (III)	1.7/1.1 (III)	0.2/0.7 (II)	0.2/− 0.3 (II/I)	0.6/− 0.2 (II/I)	2.1/1.5 (IV/III)
EF	3.8/3.5 (II)	3.6/2.9 (II)	1.3/2.2 (I)	1.3/1.1 (I)	1.6/1.2 (I)	4.5/3.9 (II)
ER	168.8/117.4 (IV/III)	27.8/16.3 (I)	4.0/4.8 (I)	2.0/1.2 (I)	12.8/6.5 (I)	7.2/4.4 (I)
< 63 μm						
*I*_geo_	2.1/1.9 (IV/III)	1.9/2.0 (III)	0.1/0.2 (II)	− 0.2/− 0.6 (I)	0.8/0.4 (II)	2.5/2.3 (IV)
EF	2.9/3.1 (II)	2.6/3.4 (II)	0.7/0.9 (I)	0.5/0.5 (I)	1.2/1.1 (I)	4.1/4.1 (II)
ER	190.0/164.6 (IV)	28.7/30.1 (I)	3.2/3.3 (I)	6.3/5.0 (I)	12.9/9.7 (I)	8.9/7.2 (I)

Classes of pollution: (i) *I*_geo_: I – unpolluted, II – unpolluted to moderately polluted, III – moderately polluted, IV – moderately to strongly polluted; (ii) EF: I – minimal, II – moderate; (iii) ER: I – low, II – moderate, III – considerable, IV – high

**Table 4:** Levels of street dust pollution according to geoaccumulation index

	Geoaccumulation index – number of samples in pollution class (2013/2018)						
	Unpolluted	Unpolluted to moderately polluted	Moderately polluted	Moderately to strongly polluted	Highly polluted	Highly to extremely polluted	Extremely polluted
63–200 μm							
Cd	0/0	0/1	36/43	7/0	1/0	0/0	0/0
Cr	11/5	31/30	2/9	0/0	0/0	0/0	0/0
Cu	1/5	4/12	24/17	13/10	1/0	1/0	0/0
Ni	34/32	10/12	0/0	0/0	0/0	0/0	0/0
Pb	3/28	35/16	4/0	2/0	0/0	0/0	0/0
Zn	0/0	3/5	13/33	26/4	2/2	0/0	0/0
< 63 μm							
Cd	0/0	0/0	22/32	22/12	0/0	0/0	0/0
Cr	2/5	41/32	1/7	0/0	0/0	0/0	0/0
Cu	0/0	1/3	17/11	22/19	4/11	0/0	0/0
Ni	2/6	40/38	2/0	0/0	0/0	0/0	0/0
Pb	0/0	20/36	23/8	1/0	0/0	0/0	0/0
Zn	0/0	0/1	4/7	30/34	10/2	0/0	0/0

**Table 5:** Levels of street dust pollution according to the enrichment factor and ecological risk factor

	Enrichment factor – number of samples in pollution class (2013/2018)					Ecological risk factor – number of samples in pollution class (2013/2018)				
	Minimal	Moderate	Significant	Very high	Extreme	Low	Moderate	Considerable	High	Very high
63–200 μm										
Cd	1/0	42/44	1/0	0/0	0/0	0/1	0/0	26/42	18/1	0/0
Cr	44/23	0/20	0/1	0/0	0/0	44/44	0/0	0/0	0/0	0/0
Cu	1/14	39/24	4/6	0/0	0/0	36/43	6/1	1/0	0/0	0/0
Ni	44/43	0/1	0/0	0/0	0/0	44/44	0/0	0/0	0/0	0/0
Pb	44/44	0/0	0/0	0/0	0/0	44/44	0/0	0/0	0/0	0/0
Zn	0/3	35/38	9/2	0/1	0/0	44/44	0/0	0/0	0/0	0/0
< 63 μm										
Cd	0/0	44/43	0/1	0/0	0/0	0/0	0/0	8/19	36/25	0/0
Cr	44/40	0/40	0/0	0/0	0/0	44/44	0/0	0/0	0/0	0/0
Cu	3/8	38/19	3/17	0/0	0/0	26/27	17/13	1/4	0/0	0/0
Ni	0/0	0/0	0/0	0/0	0/0	44/44	0/0	0/0	0/0	0/0
Pb	42/3	2/41	0/0	0/0	0/0	43/44	1/0	0/0	0/0	0/0
Zn	0/1	32/28	12/15	0/0	0/0	44/44	0/0	0/0	0/0	0/0

#### Enrichment factor

For the 63–200 μm fraction, minimal enrichment with Cr, Ni and Pb and a moderate enrichment with Cd, Cu and Zn were found. Only nine Zn samples showed significant enrichment in 2013. The level of pollution in 2018 was lower for all elements except Cr. In the case of the < 63 μm fraction, minimal enrichment occurred for Cr, Ni and Pb in 2013, moderate enrichment for Cd and Cu in 2013, Pb in 2018 and some Zn samples in 2018. Significant enrichment was found for Cu (17 samples) and Zn (15 samples) in 2013 (Tables 3, 4 and 5).

#### Ecological risk factor

For all elements except Cd, low levels of pollution (risk) occurred both in 2013 and 2018. In the 63–200 μm fraction, considerable levels of pollution were found for Cd in 2018, and considerable and high levels (18 samples) in 2013. This level was higher for the finer fraction: 36 (2013) and 25 (2018) samples showed a high level of pollution. Moderate pollution with Cu was found in the same fraction in 17 and 13 samples (in 2013 and 2018, respectively) of Cu (Tables 3, 4 and 5).

#### Potential ecological risk

For potential ecological risk in the case of the 63–200 μm fraction, in 2013, a low level of pollution was found in 3 samples, and a moderate level in 41 samples, while in 2018, in 16 and 28 samples, respectively. In the < 63 μm fraction, the level of pollution defined by this index was higher in 2013: a moderate level of pollution was found in 31 samples and a considerable level in 13 samples, while in 2018, the pollution decreased: a moderate level was found in 37 samples and a considerable level in 7 samples. Cd was the element that had the strongest influence on the level of this index.

### Statistical analyses

Principal component analysis (PCA) and cluster analysis (CA) were carried out for data normalised to the geochemical background. Such a normalisation is required due to varying natural content levels of elements. Without this statistical measure, elements with naturally high content levels would be assigned to the same group as elements whose high content levels result from anthropogenic supply. Background normalisation shows the relative content of a given element in relation to its natural content.

PCA is mainly used to generate substitute parameters that can be interpreted with a considerable number of data. It allows distinguishing groups of data with similar statistical characteristics. In the case under study, they enable distinguishing groups of elements that are presumed to be of similar origin (Han et al. 2006; Tokalıoğlu and Kartal 2006).

CA enables the determination of geometric, multidimensional Euclidean distances between the specific elements. The distances between data points were analysed using Ward’s method. The results can be used to confirm PCA results. They can also provide additional (multidimensional) information on the mutual relationships of data.

For the data from 2013, three principal components account for more than 90% of variation (Tables 6 and 7). The influence of elements on the components is also the basis for distinguishing groups of elements with similar characteristics. In the case of the 63–200 μm fraction, Cr, Cu, Zn and Cd have the biggest influence on the first component; Pb and, to a smaller degree, Ni on the second component; Ni on the third one; and Cr on the fourth one. The fourth component shows that Cr cannot be unequivocally assigned to the first group alongside Cu, Zn and Cd. The second component enables the distinction of the Pb and Ni groups, while Cr forms a separate group (Tables 6 and 7). In the case of the finer fraction (< 63 μm), Cr, Ni, Cu, Zn and Cd have the biggest influence on the first component; Pb on the second component; Ni on the third one; and Cr on the fourth one. In view of these data, Pb stands out, which is clearly shown by its influence on the second component. The first component groups the other elements together, but within this group, a subgroup of Pb and Ni can be distinguished based on the other components. Cr and Cu cannot be assigned unequivocally to any group.

**Table 6:** Fragment of the matrix of eigenvectors with four principal components for the 63–200 μm fraction

	Factor 1	Factor 2	Factor 3	Factor 4
63–200 μm fraction (2013)				
Cd	*− 0.845*	− 0.429	0.162	− 0.168
Cr	*− 0.877*	− 0.042	− 0.155	*0.444*
Cu	*− 0.861*	− 0.428	0.121	− 0.061
Ni	− 0.770	*0.358*	*− 0.466*	− 0.217
Pb	− 0.627	*0.625*	0.457	0.002
Zn	*− 0.939*	0.107	− 0.036	− 0.031
Total influence on component variation	68.279	15.005	8.219	4.637
63–200 μm fraction (2018)				
Cd	− 0.282	*0.769*	*0.316*	− 0.310
Cr	− 0.367	*0.521*	*− 0.710*	− 0.041
Cu	− 0.518	*0.453*	*0.275*	*0.655*
Pb	*− 0.735*	− 0.107	*0.312*	− 0.417
Ni	*− 0.764*	− 0.208	− 0.304	− 0.039
Zn	*− 0.674*	− 0.601	0.0460802	0.148
Total influence on component variation	34.416	24.778	14.5673	8.886

**Table 7:** Fragment of the matrix of eigenvectors with four principal components for the <63 μm fraction

	Factor 1	Factor 2	Factor 3	Factor 4
< 63 μm fraction (2013)				
Cd	*− 0.858*	− 0.294	− 0.329	0.131
Cr	*− 0.909*	− 0.050	0.058	0.232
Cu	*− 0.849*	− 0.302	− 0.176	*− 0.376*
Ni	*− 0.821*	0.014	*0.539*	− 0.119
Pb	− 0.546	*0.803*	− 0.200	− 0.099
Zn	*− 0.919*	0.112	0.049	0.161
Total influence on component variation	68.40	13.98	7.954	4.388
< 63 μm fraction (2018)				
Cd	*− 0.739*	0.325	0.232	− 0.472
Cr	− 0.011	*− 0.876*	*0.456*	− 0.027
Cu	− 0.543	− 0.492	*− 0.564*	− 0.342
Ni	− 0.825	− 0.117	− 0.082	*0.429*
Pb	*− 0.806*	0.109	*0.428*	0.002
Zn	*− 0.851*	0.054	− 0.173	0.210
Total influence on component variation	48.324	19.106	13.358	9.506

For the data from 2018, the influence of components on variation is more varied. In the case of the 63–200 μm fraction, the first three principal components account for only 73.77% of variation, and in the case of the < 63 μm fraction, for 80.79% of the variation. In the case of the 63–200 μm fraction, Ni, Zn and Pb have the biggest influence on the first component; Cr, Cu and Cd on the second one; Cr and, independently of it, Cu, Cd and Pb (inverse correlation in relation to Cr) on the third one; and Cu on the fourth one. In the case of the finer fraction (< 63 μm), Ni, Zn, Cd and Pb have the biggest influence on the first component; Cr on the second one; Cu and, independently of it, Cr and Pb on the third one; and Ni on the fourth one (Tables 6 and 7).

**Fig. 4:**
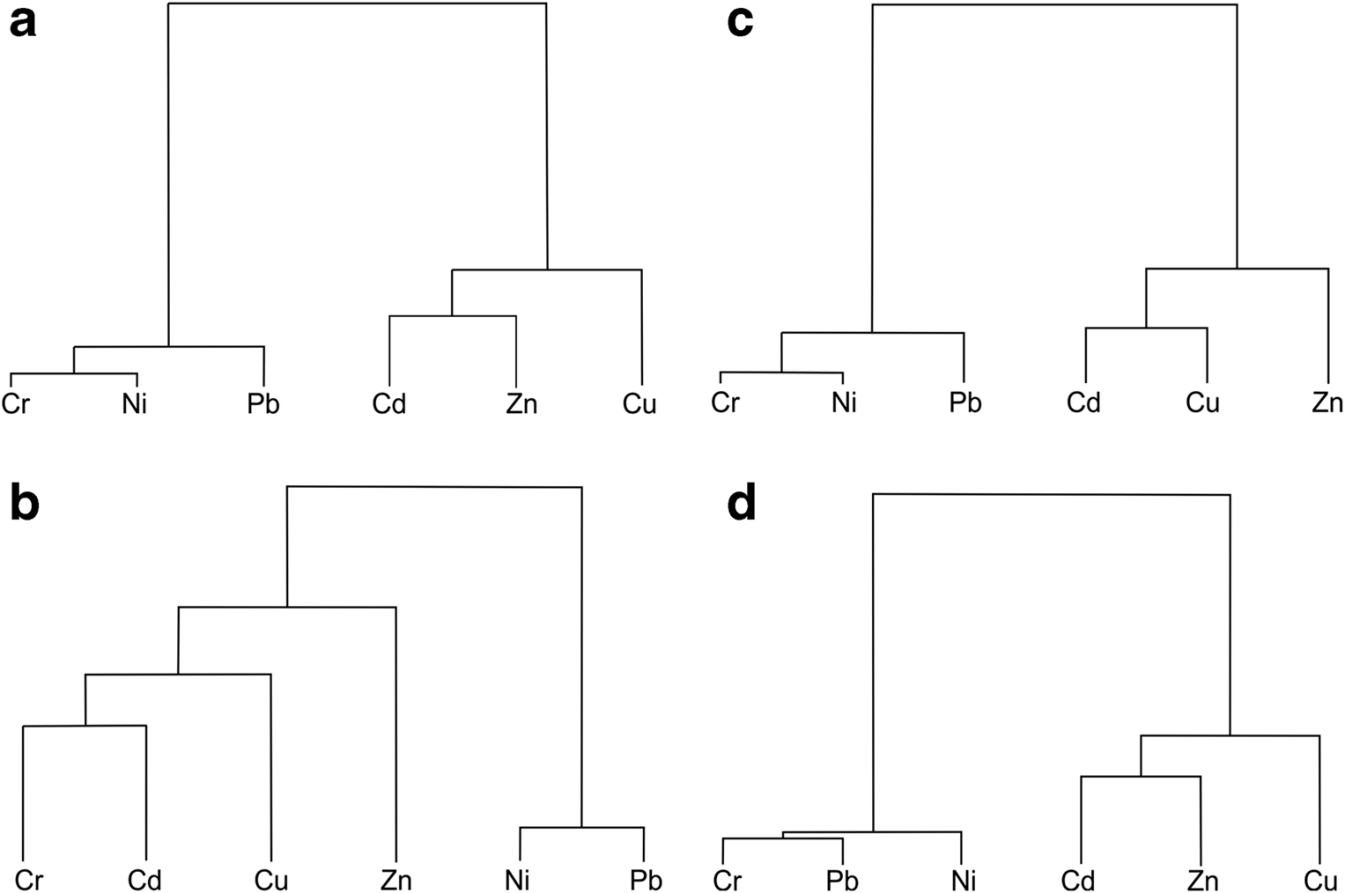
Results of grouping, using Ward’s method, for data normalised to the background values. (A) 63–200 μm fraction, 2013. (B) 63–200 μm fraction, 2018. (C) < 63 μm fraction, 2013. (D) < 63 μm fraction, 2018

The results of cluster analysis indicate the existence of two groups of elements for both fractions under study in 2013: (i) Cr, Ni and Pb and (ii) Cd, Zn and Cu. Zn constitutes a separate group for the 63–200 μm fraction in 2018 (Fig. 4.)

## Discussion

### Levels of pollution

Heavy metal content in street dust and upper soil horizons in cities is an index of pollution originating from various forms of human activity—operation of industrial plants, fuel consumption, exhaust emissions and the wear of various vehicle parts (Al-Khashman 2013; Bilos et al. 2001). In a case of Lublin, the highest degree of enrichment in relation to the geochemical background is shown by Zn, Cu and Cd. The pollution of street dust with Zn is linked with, above all, the wear of the moving car parts (Al-Khashman 2004). Cu, on the other hand, is commonly used in motor vehicle oils (Al-Khashman 2007). It can also originate from the wear of motor oil pumps and corrosion of metal car parts (De Miguel et al. 1997) as well as engine wear and tear (Al-Khashman 2007; Jaradat and Momani 1999). The results of studies conducted by Plak et al. (2010) indicate that higher Cu concentrations in the soils in Lublin result from the use of copper overhead wires transmitting electrical energy to trolleybuses. Increased Cd levels in street dust are linked with motor vehicle traffic (brake lining components) and combustion of coal (Divrikli et al. 2005).

The concentrations of heavy metals (except Cd) found in studied street dust in Lublin were distinctly lower than those in other cities of the world (Table 8). This results from the fact that Lublin is a relatively small city where, at present, big industrial plants do not exist, while road traffic intensity is not very high. Therefore, the level of environmental pollution in Lublin is lower. In addition, some studies, mentioned in Table 8, describe the conditions from 10 or 20 years ago; hence, a straightforward comparison is not possible. We also compare our results with the data for Dhaka (Bangladesh) obtained by means of the same method and equipment (Epsilon5 Panalytical). The mean concentrations of heavy metals in Lublin were generally lower for Cd, Cr and Ni, but the level of pollution in a case of Cu, Pb and Zn in 2013 was similar (Safiur Rahman et al. 2019).

**Table 8:** Heavy metal content in street dust fractions according to various authors (mg kg^−1^)

Country	Year	Size (μm)	Cd	Cu	Pb	Zn	Source
Scotland	1998	< 63	1.7	500	1265	1070	Deletic and Orr (2005)
		63–250	0.6	325	305	345	
Korea	2006	< 75	2.3	345	223	1271	Duong et al. (2006)
		75–180	1.0	236	118	752	
Malaysia	2013	< 63	0.7	84	88	394	Han et al. (2014)
		63–125	0.6	50	40	243	

It should be mentioned that heavy metals stored in street dust not only pollute the soil and air of urban areas but also accumulate in alluvia and constitute a threat for the hydrosphere. They are washed away during rainfall events and accumulate in water sediments in the Bystrzyca river valley, downstream of the city (Zgłobicki et al. 2016a, b). Street dust may be considered as one of the main factors of such pollution.

### Impact of particle size

Conducted studies indicate that heavy metal concentrations decrease with the increase of particle size, and the highest content levels occur in the finest fractions (Stone and Marsalek 1996; Birch and Scollen 2003; Zafra et al. 2011; Adamiec et al. 2016; Lanzerstorfer 2018). It should be remembered, however, that the < 63 μm fraction contains less than 10% of the total amount of metals in street dust (Stone and Marsalek 1996; Birch and Scollen 2003). On the other hand, smaller particles are easily re-suspended and constitute bigger health threat (Nicholson 1988). The relationships between heavy metal content in the < 63 μm fraction and the 63–200 μm fraction of street dust in Lublin were different for the particular elements. The highest degree of enrichment of the finest fraction was found in samples from the year 2018 for Cu (2.1), Pb (1.8) and Zn (1.7) (Fig. 3). These indices were slightly lower for samples from the year 2013 and corresponded to the levels for street dust reported by Zafra et al. (2011): Cd, 1.7; Cr, 6.7; Cu, 31; Ni, 1.36; Pb, 1.25; Zn, 1.57. The smallest difference between content levels in the < 63 μm and 63–200 μm fractions occurred for Cd and Cr.

### Changes in pollution levels

According to the data from the Provincial Inspectorate of Environmental Protection in Lublin, heavy metal content levels in air decreased: Cd and Pb levels were about 1.6 times higher in 2013 than in 2018, while Ni levels were 3.3 times lower. Similar patterns have been found in the street dust samples under study. The mean ratios of concentrations in 2013 to concentrations in 2018 are as follows: (i) 63–200 μm fraction: Cd, 1.34; Cr, 0.7; Cu, 1.38; Ni, 1.36; Pb, 1.71; Zn, 1.34; (ii) < 63 μm fraction: Cd, 1.14; Cr, 0.96; Cu, 0.95; Ni, 1.25; Pb, 1.34; Zn, 1.22. The above data show that the decrease of pollution in 2018 was more distinct for the coarser fraction, and the biggest in the case of Pb. For Cr, however, content levels slightly increased in both fractions (the increase was bigger for the 63–200 μm fraction), and in the case of Cu, in the finer fraction. The reasons for the small increase of Cr content in street dust are not clear. Cr is an element of many car parts (metal plating, wrist pins and connecting rods) and its presence in street dust may be a result of more intense abrasion. Tannery industry is reported to be an important source of the element (Safiur Rahman et al. 2019) but it is not a case of Lublin. The issue requires further investigations.

For geoaccumulation index and enrichment factor (mean values), the pollution indices have a similar order: Zn > Cd > Cu > Pb > Cr > Ni (both fractions, years 2013 and 2018). This order is slightly different for the ecological risk factor: Cd > Cu > Pb > Zn > Cr > Ni. The elements under study form two distinct groups: Zn, Cd and Cu (moderate pollution) and Pb, Ni and Cr (no pollution or minimal pollution) (Table 3). Pollution is considerable or high for Cd only in the case of the ecological risk factor.

In the case of analyses taking into account the road traffic intensity (type of the road) in the 2013–2018 period, it was found that a distinct decrease for the 63–200 μm fraction within main roads occurred for Cd and Cu (Table 9). The scale of changes for the other elements was similar for both kinds of roads. At the same time, there was an approximately 10% increase in the number of registered cars during the period considered. There are two possible reasons for this situation—decrease of pollution. Supply of Cd and Cu related to the wear of brakes, tyres and other car parts was reduced. This may be due to the improvement of the technical condition of cars moving along main roads. The second option is that content of Cd and Cu in street dust is not directly related to the motor traffic. Such justification may support the results of previous studies, which did not show a connection between the content of heavy metals and the intensity of traffic (number of cars crossing) in a given place (Zgłobicki et al. 2018). A large decrease occurred for Pb in the 63–200 μm fraction for both types of roads. No correlation with car traffic was found, which indicates a decreased volume of supply from many anthropogenic sources. Ewen et al. (2009) arrived at similar conclusions when studying street dust in Thessaloniki. One of the main components of street dust is the material coming from the soil adjacent to the roads (Gunawardana et al. 2012). Perhaps the observed decrease in the content of heavy metals is caused by the decrease in soil pollution in Lublin.

**Table 9:** Changes in metal content levels in street dust in Lublin depending on vehicle traffic intensity

	Mean concentration in 2013/mean concentration in 2018					
	Cd	Cr	Cu	Ni	Pb	Zn
63–200 μm						
Main roads	1.6	0.8	2.1	1.4	1.8	1.5
Side streets	1.2	0.7	1.3	1.4	1.7	1.4
< 63 μm						
Main roads	1.1	0.9	0.9	1.2	1.4	1.3
Side streets	1.2	1.0	1.0	1.2	1.3	1.2

### Sources of pollution

Based on PCA and CA results, it can be observed that the analysed elements form groups of different origins. In 2013, one group consisting of Cu, Zn and Cd may be distinguished, i.e. elements of anthropogenic origin. The second group consisted of Pb and Ni, i.e. elements whose levels were similar to the background and mainly of natural origin. Cr content levels are determined by other factors which make these levels very unstable; hence, this element cannot be assigned to any group.

While the natural element group (Pb, Ni) did not change in 2018, the anthropogenic element group is not as distinct in statistical analysis results as it was in 2013. This results from the lower and more varied content of metals previously regarded as anthropogenic, i.e. from the “removal” of these elements from street dust. Due to the lack of distinctly anthropogenic supplies in the entire study area, this group yields ambiguous results when the results of statistical analyses (PCA and CA) are considered together.

The results of the study indicated low level of street dust pollution with heavy metals and its decrease in Lublin comparing situation in 2013 and 2018. This is probably related to the reduced emission of industrial, communication and municipal pollutants as a result of application of greener technologies (Pacyna et al. 2009; Harmens et al. 2013). The concentrations of these elements across Europe have reduced over the last decades but in the case of some industrial cities, opposite trends were also observed (Ordóñez et al. 2015). Poland is still one of the largest emitters of pollutants such as Pb, Cd and Hg, but due to the lack of large industrial plants in Lublin, there was a decrease in the volume of metal content in the road dust during the last 5 years. A further clear decrease in Pb content in the dust may also be due to the weakening effect of “old” lead that was accumulating in the soil in the period when it was used in fuel. A detailed spatial analysis carried out for Lublin indicates that the highest Cd content was found in the vicinity of the main thoroughfare. Power lines used by trolleybuses, in turn, are the source of Cu, while higher concentrations of Zn were found in the eastern part of the city, the former and present industrial district. This confirms the results of previous research (Zgłobicki et al. 2018).

### Risks

The decrease in the level of pollution, with the simultaneous increase in the number of cars, indicates that car traffic is not the main source of metals to street dust or the technical condition of cars has improved and their supply of metals has been reduced. This issue requires further study. Perhaps an important role here is played by the decline in soil contamination as a reservoir of heavy metals in the urban environment. Although it should be remembered that in the case of Lublin, road dust is currently a source of soil contamination and not the other way around (Zgłobicki et al. 2018). The combustion of low-quality coal and other energy resources in households may be an important cause of environmental pollution in Lublin. Despite the increased Zn and Cu levels found in the street dust, it seems that the greatest risk to health is the presence of elevated concentrations of highly toxic Cd. Increased levels of this element in river sediments downstream from Lublin indicate that the city is an important source of cadmium (Bojakowska and Sokołowska 1996; Zgłobicki et al. 2016a, b). One should also look at the changes in the environment of the studied area and their impact on environmental geochemistry in a broader context. The conducted research indicates that the soils and river sediments in the vicinity of Lublin generally show a low degree of pollution (Lis and Pasieczna 1995; Zgłobicki and Rodzik 2007; Zgłobicki et al. 2011), while the increasing forest cover (Zgłobicki et al. 2017) leads to the improvement of atmospheric air quality. This allows us to assume that the risk to human health associated with the presence of heavy metals in the street dust will be reduced.

## Conclusions

We found that the levels of street dust pollution with heavy metals, expressed both in absolute concentrations and geochemical concentrations, were lower in 2018 than those in 2013. The biggest decrease occurred in the case of Pb; the decrease for Ni and Zn was slightly lower, and the one for Cd and Cu was the lowest. The content of Cr slightly increased.

For 2013 and 2018, metals in both street dust fractions are in the following order in terms of enrichment in relation to the background: Zn > Cd > Cu > Pb > Cr > Ni. Cu and Zn are the predominantly anthropogenic elements. In the case of the other elements, the influence of supply related to human activity is distinctly lower; it is the lowest for Cr and Ni.

Higher concentrations of metals occurred in the < 63 μm fraction. This pattern was particularly visible for Pb, Cu and Zn. In the case of Pb and Cu, a distinctly higher content in the finer fraction was observed in 2018. This fraction is susceptible to re-suspension and thus poses a greater threat to human health, which is further aggravated by higher levels of heavy metal content. What is more, the decrease in 2018 was less intensive for this fraction.

For some metals, a correlation was found between motor vehicle traffic and changes in their concentration levels in street dust. The intensity of changes also depended on the fraction. The clearest decrease of concentration levels occurred within the main roads, in the 63–200 μm fraction for Cu and Cd, and in both fractions for Pb.

Heavy metals in Lublin’s road dust do not pose a significant health risk. Only the presence of elevated levels of toxic Cd can be alarming and requires further detailed studies. They should primarily concern the sources of this element in the urban environment.
